# Arteriovenous Malformation of the Cervical Spine Presenting as Subarachnoid Hemorrhage

**DOI:** 10.7759/cureus.7200

**Published:** 2020-03-07

**Authors:** Travis M Cox, Daniel M Chavez Andia, Gabriel Aisenberg

**Affiliations:** 1 Internal Medicine, University of Texas John P. and Kathrine G. McGovern School of Medicine, Houston, USA; 2 Internal Medicine, St. Luke's Hospital, St. Louis, USA

**Keywords:** arteriovenous malformation, cervical, headache

## Abstract

Arteriovenous malformations (AVM) of the cervical spine can present with symptoms resulting from their mass effect, vascular steal, or subarachnoid hemorrhage (SAH). While ruptured cerebral aneurysms bleed fast and usually cause severe headache, AVM bleed slowly; moreover, when the location is extracranial, the presentation might be even more confusing. For these reasons, the clinical course can be misleading. We present the case of a woman who had bleeding from an AVM of the cervical spine and discuss the classification and treatment options of AVM.

## Introduction

Arteriovenous malformations (AVM) of the cervical spine can present with symptoms resulting from their mass effect, vascular steal, or subarachnoid hemorrhage (SAH) [1]. Ruptured cerebral aneurysms bleed fast and usually cause severe headache. AVM bleeds slowly; yet, when the location is intracranial, the presentation might resemble that of bleeding aneurysms, whereas extracranial locations can make the clinical course confusing.

We discuss here the case of a woman who came to the hospital with two complaints: chest pain consistent with acute coronary syndrome and headache for four days. The workup for the chest pain led to normal findings. Further evaluation of the headache, given its persistent nature and subsequent development of sensorimotor deficits, led to the diagnosis of a spinal hematoma secondary to a cervical AVM.

## Case presentation

A 59-year-old woman presented to the emergency department with chest pain radiating to the left shoulder, arm, and back, and two days of headache. Her chest pain virtually resolved on admission, and the workup for acute coronary syndrome was negative. Her headache, however, fluctuated in intensity, and became worse on the third day, when she also reported some numbness on her left fingertips. A computed tomography (CT) of the head was normal.

Her blood pressure was 157/68 mmHg, pulse 67/minute, respiratory rate 18/minute, temperature 36.2ºC. Her neck was supple, with no paraspinal tenderness. There was no focal neurologic deficit.

**Figure 1:**
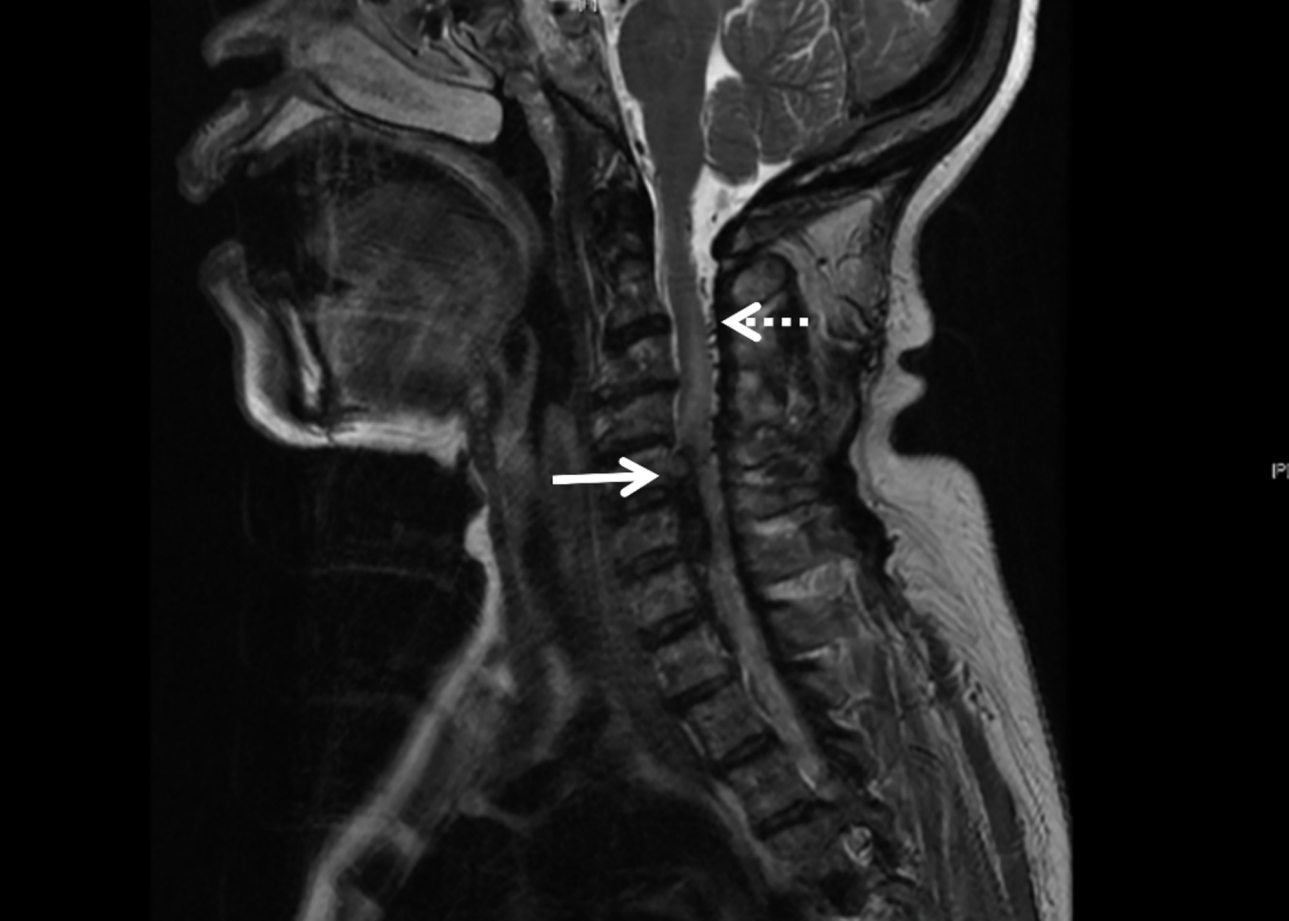
Sagittal magnetic resonance imaging (MRI) shows a C5-C6 spinal hematoma (solid arrow), and abnormal vessels adjacent to the dorsal aspect of the cervical canal (dotted arrow)

**Figure 2:**
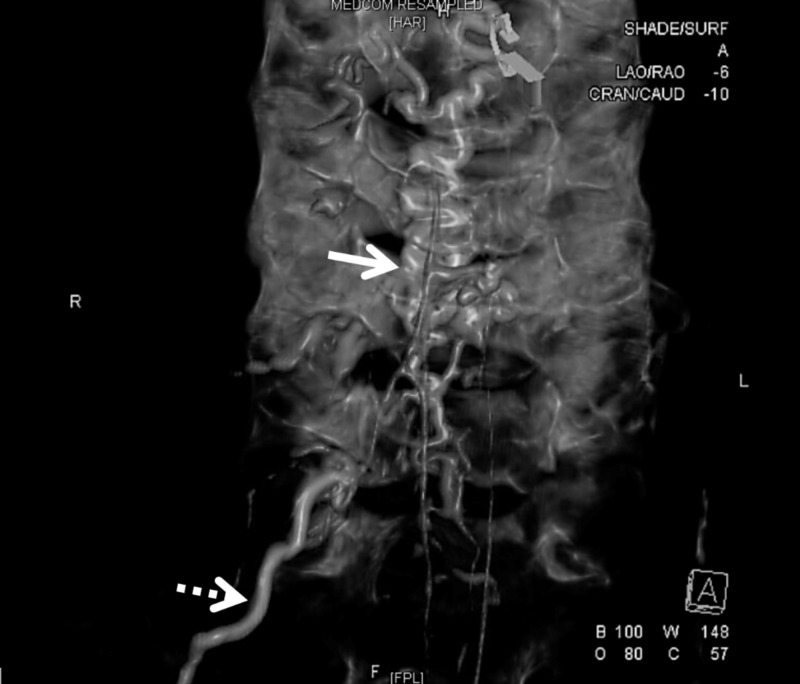
Angiogram shows the arteriovenous malformation nidus (solid arrow) supplied by the anterior spinal artery and a radicular branch of the right vertebral artery (dotted arrow), where the contrast was delivered

On the fourth day, the headache level increased. Her paresthesia level was reportedly more proximal, affecting the left forearm, and she developed difficulty with flexion and abduction of that arm. No deep reflexes were elicited on that limb. A lumbar puncture performed under radiologic guidance produced a bloody fluid. Its analysis showed 2,440,000 red blood cells/μL, 1,100 white blood cells/μL (8% neutrophils, 56% lymphocytes, 36% macrophages), glucose 14 mg/dL, protein level of 376 mg/dL, and negative Venereal Disease Reseach Laboratory (VDRL) test. Magnetic resonance imaging (MRI) showed a C5-C6 spinal hematoma and abnormal vessels adjacent to the dorsal aspect of the cervical canal (Figure 1). An angiogram showed the arteriovenous malformation nidus (type 4) supplied by the anterior spinal artery and a radicular branch of the right vertebral artery (Figure 2). The patient underwent a cervical laminectomy. The hematoma was evacuated. The AVM was dissected, coagulated, and resected. Postoperatively the patient improved substantially with minimal residual left arm weakness six months after discharge. 

## Discussion

AVMs cause less than 5% of all SAH [1]. Most physicians are better acquainted with stereotypical subjective features of SAH resulting from ruptured aneurysms, such as severe (“worst ever”) and abrupt presentation of headaches, sometimes associated with nausea, vomiting, syncope, visual disturbances, or seizures. These symptoms result from the rapid accumulation of arterial blood in the subarachnoid space. This is in stark contrast with SAH caused by spinal AVM, which often presents with paresis as the initial symptom, and by the time of diagnosis, with back pain, pain originated in the nerve roots, sensory changes, paresis, and bowel or bladder dysfunction. When paresis is present, it usually affects the lower limbs due to the more frequent presentation of AVM in the thoracic or lumbar spine than in the cervical spine [2]. Unlike spinal AVM, those with intracranial location can present with similar symptoms as those from ruptured aneurysms, though usually in a slower, vaguer manner. The capacity of the spinal subarachnoid space allows for larger blood accumulation, delaying the symptom appearance. Considering the reported re-bleeding rate of 6%-15%, the early recognition of an AVM may impact clinical outcomes [3].

The classification of AVM has sustained multiple changes. Currently, type IV AVM represents those with a vascular nidus that involves the spinal parenchyma with or without the involvement of an axial artery or vein [4]. Overall, AVM more commonly causes intracranial bleeding than spinal bleeding; the different classes are not associated with a known differential bleeding risk [5]. AVM can cause symptoms as a result of their mass effect, vascular steal, or hemorrhage. In the end, it is the clinical manifestation and the type of lesion that determine the best therapeutic strategy [6]. In this case, a combination of decompression and AVM ablation were indicated.

## Conclusions

SAH can sometimes present with an indolent course, especially when it results from low-flow lesions, like AVM, particularly those with extracranial location, such as the spinal subarachnoid space that has a larger capacity for blood accumulation. A non-resolving or progressive headache, with or without neurologic deficit, should raise suspicion for such presentation.
